# Chikungunya Fever and Rheumatoid Arthritis: A Systematic Review and Meta-Analysis

**DOI:** 10.3390/tropicalmed10020054

**Published:** 2025-02-12

**Authors:** José Kennedy Amaral, Robert Taylor Schoen, Michael E. Weinblatt, Estelita Lima Cândido

**Affiliations:** 1 Postgraduate Program in Health Sciences, Faculty of Medicine, Federal University of Cariri, 284 Divino Salvador Street, Barbalha 63090-000, Ceará, Brazil; estelita.lima@ufca.edu.br; 2 Section of Rheumatology, Allergy, and Immunology, Yale University School of Medicine, 15 York Street, New Haven, CT 06510, USA; robert.schoen@yale.edu; 3 Division of Rheumatology, Inflammation and Immunity, Harvard Medical School, Brigham and Women’s Hospital, 60 Fenwood Road, Boston, MA 02115, USA; mweinblatt@bwh.harvard.edu

**Keywords:** chickungunya fever, rheumatoid arthritis, joint disease, autoimmune arthritis

## Abstract

Chikungunya fever (CHIKF) is a re-emerging infectious disease caused by the chikungunya virus (CHIKV), transmitted primarily by Aedes mosquitoes. A significant number progress to chronic chikungunya arthritis, which shares similarities with rheumatoid arthritis (RA). Despite evidence of a link between CHIKV infection and subsequent RA development, a comprehensive analysis of the relationship between these two diseases is lacking. This study systematically analyzes the incidence of RA after CHIKV infection and its immunological mechanisms, following PRISMA guidelines with literature searches across multiple databases up to 3 September 2024. Eligible studies included retrospective and prospective designs reporting RA diagnoses after CHIKV infection. Data extraction was performed independently, and the risk of bias was assessed using appropriate tools. Sixteen studies involving 2879 patients were included, with 449 individuals diagnosed with RA following CHIKV infection, resulting in a combined incidence of 13.7% (95% CI: 6.12% to 27.87%). High heterogeneity between studies was observed (*I*^2^ = 96%), indicating variability related to diagnostic criteria and population characteristics. This review highlights the significant RA incidence after CHIKV infection, emphasizing the need for research on autoimmune mechanisms, long-term rheumatological follow-up, early diagnostic biomarkers, and CHIKV’s long-term health impacts.

## 1. Introduction

Chikungunya fever (CHIKF) is a mosquito-borne, viral infection that has re-emerged in global outbreaks over the past 75 years. First reported in 1952, CHIKF has since spread to over 100 countries, with over 4 million cases reported, particularly after the first case in the Americas in 2013 [1,2]. The chikungunya virus (CHIKV), a positive-sense single-stranded RNA *Alphavirus* virus is transmitted by *Aedes mosquitoes* (*Ae. aegypti* and *Ae. albopictus*) [3]. The disease presents with sudden fever, incapacitating polyarthritis, myalgia, rash, headache, and gastrointestinal symptoms, typically 2 to 6 days after infection [4]. While usually self-limiting, with recovery in 10 days, up to 40% of cases progress to a chronic phase, chronic chikungunya arthritis (CCA), characterized by persistent or deforming arthritis [5].

Following the global spread from Africa of the vectors of CHIKV, *A. aegypti* and *A. albopictus*, and the increase in international travel, there has been rapid geographic expansion of CHIKV infection. With the resultant increasing CCA cases, better characterization of the clinical features of CCA has been possible [6,7]. Within the clinical spectrum of CCA, some patients present as RA “mimics” with symmetric polyarthritis, morning stiffness, frequent hand involvement, and in some individuals, positive RA biomarkers. [8,9,10]. Not infrequently, these individuals meet ACR (American College of Rheumatology)/EULAR (European Alliance of Associations for Rheumatology) classification criteria for RA [11,12] Whether CCA mimics RA or whether CHIKV infection increases the risk of developing ongoing RA is still a subject of debate.

The similarities between CCA and RA are pathological as well as clinical [5,10,11,13]. RA is a systemic inflammatory disease of multifactorial etiology characterized by chronic and progressive synovitis [9,10,11]. RA pathogenesis involves interactions between genetic, environmental, and immunological factors, resulting in dysregulated immune responses and chronic inflammation, damaging joints and other organs [10]. Immunophenotyping of peripheral blood mononuclear cells in RA and CCA reveals a common inflammatory profile, suggesting shared immunological mechanisms. Viral persistence in joints, detection of viral genetic material in the synovium, and activation of innate and adaptive immune responses are mechanisms proposed to explain CCA pathogenesis. Autoimmunity induced by molecular mimicry or virus-induced tissue damage has also been considered [8,11].

There has long been interest in whether RA is linked to viral infection [12]. For CCA, there is a fundamental pathogenic question as to whether the arthritis phase results from persistent viral infection or a post-viral inflammatory process [13,14]. There is also a question as to whether CCA patients benefit from a disease-modifying treatment strategy, borrowed from the treatment of RA [11]. For these multiple reasons, we conducted a systematic review and meta-analysis to evaluate current evidence on the development of RA following CHIKV infection. We believe that the increasingly available data justify further investigation of pathogenic similarities between these two diseases and support CCA therapeutic strategies informed by RA treatment.

## 2. Materials and Methods

The study protocol was registered in the International Prospective Register of Systematic Reviews (PROSPERO) (protocol CRD42024585798). This study was conducted according to the Preferred Reporting Items for Systematic Reviews and Meta-Analyses (PRISMA) [15] guidelines to address the following question: “What are the characteristics and incidence of rheumatoid arthritis development in patients after chikungunya virus infection?”

### 2.1. Information Sources

We systematically searched PubMed, Cochrane Library, Scielo, LILACS, Web of Science, and Scopus for relevant literature published until 3 September 2024. We also incorporated studies found through manual searches using Google Scholar, gray literature, and reference lists. Articles in English, Spanish, or Portuguese were included.

### 2.2. Eligibility Criteria

Retrospective and prospective studies, randomized controlled trials, and case series reporting diagnosis of RA after CHIKV infection, with clinically and laboratory-confirmed testing for anti-CHIKV-specific immunoglobulin M (for acute cases) or immunoglobulin G (post-exposure) detected by enzyme-linked immunosorbent assay (ELISA) or RNA virus by reverse-transcriptase polymerase chain reaction were included. Studies were excluded if they did not include laboratory confirmation of CHIKV, did not follow the ACR-EULAR classification criteria, or presented insufficient data for analysis. We excluded from this systematic review: letters to the editor, editorials, review articles, commentaries, preclinical trials, and papers without relevant data.

### 2.3. Search Strategy

Search terms included combinations of free-text and MeSH or Emtree terms for CHIK and RA, including (“Chikungunya virus” (MeSH) OR “Chikungunya fever” (MeSH) OR “CHIKV” OR “Chikungunya infection” OR “Chikungunya” OR “CHIK” OR “CHIKV”) AND (“Arthritis, Rheumatoid” (MeSH) OR “Rheumatoid arthritis” OR “Autoimmune arthritis” OR “RA”). Information about the databases used, search dates, search methods, and the number of studies retrieved is provided in the Supplementary Materials (Table S1).

### 2.4. Selection Process

Two independent reviewers (J.K.A. and R.T.S.) screened titles and abstracts based on the eligibility criteria. Studies with uncertain relevance were included to ensure all potentially relevant work was considered. Disagreements were resolved through discussion, with a third reviewer involved if necessary. The same process was applied to full-text reviews, with further evaluation of studies with unclear relevance at the abstract stage.

### 2.5. Data Collection Process

Independent reviewers (J.K.A. and R.T.S.) extracted data using a standardized form (Table S2). Afterward, a second reviewer independently verified the data to ensure all relevant information was included. Any discrepancies were resolved through discussion, with a third reviewer involved if necessary. The form captured details such as author, publication year, country, study design, patient demographics, type of chikungunya diagnosis, number of RA cases, comparators, evaluation measures, symptoms, clinical progression, and study results.

### 2.6. Data Items

Data were collected on the following outcomes: the incidence of RA, defined by the percentage of patients who developed RA after CHIKV infection, as diagnosed by the authors of the included studies using standard clinical and laboratory criteria; the clinical and demographic characteristics of patients, including mean age (±standard deviation), sex (male/female), country of origin, and type of chikungunya diagnosis (acute or post-exposure), as determined by the detection of anti-CHIKV IgM or IgG antibodies or viral RNA by PCR; the measures used to assess RA, such as clinical examinations, laboratory tests, and established diagnostic criteria; the clinical evolution of patients, including descriptions of symptoms, duration of arthritis, clinical progression of the disease, and response to specific treatments, when applicable; and other relevant outcomes, including any other relevant findings associated with the development of RA following CHIK infection, as reported in the included studies.

### 2.7. Study Risk of Bias Assessment

The risk of bias in the included studies was assessed using tools appropriate to each study design, focusing on those that confirmed CHIKV infection through serological tests (IgM/IgG anti-CHIKV) or other validated methods. Two reviewers independently evaluated the risk of bias using the Newcastle–Ottawa Scale (NOS) for cohort studies, the Joanna Briggs Institute (JBI) Critical Appraisal Checklist for case series and observational studies, and the RoB 2.0 tool for randomized controlled trials [16,17,18,19].

## 3. Results

### 3.1. Study Selection

**Figure 1:**
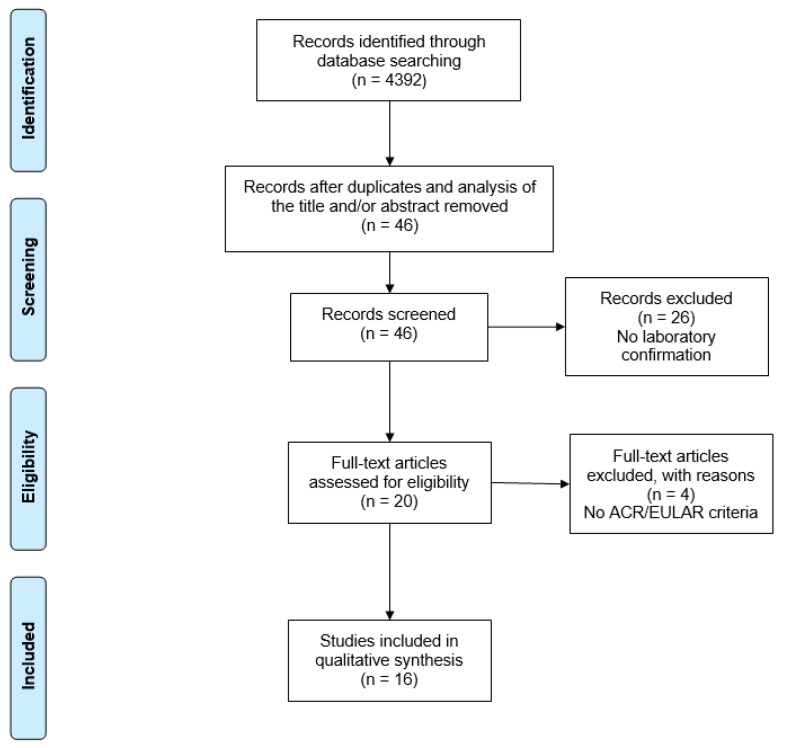
PRISMA flow diagram of search results. PRISMA: Preferred Reporting Items for Systematic Reviews and Meta-Analyses.

A total of 4392 studies were retrieved from the database searches. After exclusion by reading the titles and/or abstracts and duplicates, only 46 articles were selected for full reading. Sixteen studies met the eligibility criteria and were included in the systematic review (Figure 1).

### 3.2. Study Characteristics

The studies included in this systematic review were conducted in diverse geographical and epidemiological settings, reflecting the wide geographic distribution of CHIKV infection and its rheumatological consequences. Two cross-sectional studies were conducted in Brazil [20] and Colombia [21]. Nine cohort studies included research from Bangladesh [22], the United States [23], France [24], India [25,26,27], Colombia [28,29], and Reunion Island [30]. Four case series studies were conducted in the United States [31], France [32], Reunion Island [33], and Brazil [34]. The randomized controlled trial was conducted in India [35].

### 3.3. Risk of Bias in Studies

#### 3.3.1. Cross-Sectional and Case Series Studies

Among the cross-sectional studies, both Hayd et al. [20] and Tritsch et al. [21] were rated as having a moderate risk of bias. These studies showed limitations in controlling for potential confounding factors that could affect the validity of their findings. Similarly, among the case series studies, there was variable bias risk. While some case series, such as Javelle et al. [33], were rated with low risk of bias due to their clear inclusion criteria and consistent measurement of outcomes, others, including Miner et al. [31], Bouquillard and Combe [32], and Amaral et al. [34], were assessed as having a moderate risk of bias due to concerns related to participant selection and the handling of incomplete data.

#### 3.3.2. Cohort Studies

A significant proportion of the cohort studies (n = 7 of 9) were rated with low risk of bias. These studies, such as Pollett et al. [23], Manimunda et al. [25], Segura-Charry et al. [28], Rodriguez-Morales et al. [29], Paul et al. [26], Mathew et al. [27], and Guillot et al. [30], adhered to rigorous methodological standards, including valid and reliable measurement of exposures and outcomes, identification and control of confounding factors, and sufficient follow-up duration to capture relevant outcomes.

Two cohort studies were rated with a moderate risk of bias. Hossain et al. [22] and Bouquillard et al. [24] had incomplete follow-up and inadequate handling of confounding factors that could introduce bias into their findings.

#### 3.3.3. Randomized Controlled Trials

The randomized controlled trial by Ravindran and Alias [35] was rated with some bias concerns using the RoB 2.0 tool. The study was an open-label design, raising potential bias in the measurement of subjective outcomes. Although the trial demonstrated strength in areas like randomization and handling of missing data, the open-label nature could have influenced participant behavior or reporting, leading to “some concerns” about the overall risk of bias.

The details are reported in the Supplementary Materials.

### 3.4. Results of Individual Studies

A total of 2879 patients infected by CHIKV were found in the included studies, with 449 diagnosed with RA. The studies differed by location and methods but consistently identified patients meeting RA diagnostic criteria after infection. Common symptoms included persistent joint pain, morning stiffness, polyarthralgia, joint swelling, and auto-antibodies such as rheumatoid factor (RF) and anti-cyclic citrullinated peptide (anti-CCP). Severe cases showed bone erosions, synovitis, and required advanced therapies like tumor necrosis factor (TNF) blockers and other biologics.

For example, Segura-Charry et al. [28] reported that 87 of 410 patients developed RA, with many requiring biologics therapy due to disease severity. In Guillot et al. [30], 40 of 159 patients had persistent symptoms up to 13 years post infection, highlighting the chronic nature of RA after CHIKV. Bouquillard and Combe [32] and Javelle et al. [33] demonstrated the effectiveness of methotrexate (MTX) in alleviating symptoms and slowing disease progression. Ravindran and Alias found combination therapy with MTX, sulfasalazine, and hydroxychloroquine (HCQ) more effective than HCQ monotherapy in reducing RA activity after CHIKV [35].

See Table 1 for details on each study, including diagnosed cases and clinical characteristics.

### 3.5. Quality Assessment

Most of the studies included in the systematic review demonstrated good methodological quality, with low or moderate risk of bias. The rigorous application of the NOS, JBI, and RoB 2.0 bias assessment tools allowed for a clear identification of the strengths and limitations of each study.

### 3.6. Meta-Analysis

**Figure 2:**
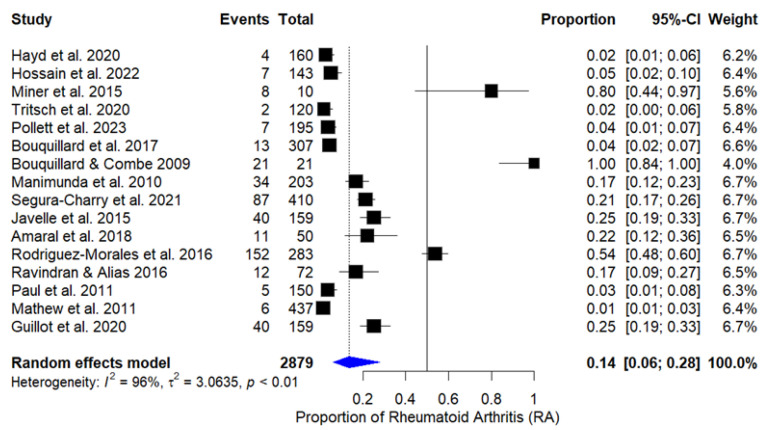
Forest plot of proportion of rheumatoid arthritis in patients after chikungunya infection [20,21,22,23,24,25,26,27,28,29,30,31,32,33,34,35].

The combined proportion of patients who developed RA after CHIKV infection was 13.7% (IC 95%: 6.12% to 27.87%), based on the random effects model, due to high heterogeneity among studies (*I*^2^ = 96%, *Q* = 373.00, *p* < 0.0001). The graph in Figure 2 presents the individual estimates of RA proportion for each study, as well as the combined estimate obtained through the random effects model. After excluding cross-sectional studies and case series from the meta-analysis, heterogeneity reduced from 96% to 76.8% (*Q* = 33.4969, *p* < 0.0001), indicating better homogeneity among the remaining studies.

The analysis only with cohort studies classified as having low risk of bias resulted in an incidence of rheumatoid arthritis of 17.7% (95% CI: 4.2–31.3%). Although heterogeneity decreased compared to the full meta-analysis, it still remained high (*I*^2^ = 81.07%).

The certainty of the evidence was assessed using the GRADE approach, considering risk of bias, inconsistency, imprecision, publication bias, and applicability of the results. For the incidence of RA post CHIKV, the certainty was considered moderate, downgraded due to inconsistency (*I*^2^ = 96%, IC 95%: 6.12–27.87%).

A forest plot showing the proportions of patients who developed rheumatoid arthritis after chikungunya infection in each study is included in the meta-analysis. The diamond at the bottom represents the combined proportion with a 95% confidence interval. The random effects model was used due to the high heterogeneity observed.

## 4. Discussion

This systematic review is, to our knowledge, the first to explore the relationship between CHIKV infection and the subsequent development of RA. Despite the heterogeneity of RA incidence among these studies, the meta-analysis suggests that a significant proportion of patients develop RA after CHIKV infection. However, the observed variability indicates that factors such as population characteristics, diagnostic methods, and follow-up duration may influence results. Our systematic review was restricted to studies that assessed RA as recognized in ACR-EULAR classification criteria [11]. Rodríguez-Morales et al. conducted a more general review in which they classified RA as part of a group called “post-CHIK chronic arthritis” that also included post-viral or nonspecific arthritis and seronegative spondylitis, finding a prevalence of 13.66%. However, they did not specifically evaluate RA separately [36].

Despite the variation in follow-up time among the studies in this review, we suggest that a longer period of time may be necessary to identify RA patients after CHIKV infection, as demonstrated in the study by Guillot et al. [30]. We acknowledge that the absence of a standardized observation period among the studies may have influenced the variability of the estimates, representing a limitation of this analysis.

Many CCA patients develop RA symptoms that clinically resemble classic RA, such as persistent joint pain, morning stiffness, polyarthralgia, and joint swelling [37,38,39]. But is this RA or an RA “mimic”? Many of these patients had severe disease, with joint erosions and synovitis, suggesting that CHIKV may cause the same inflammatory processes associated with RA, as described in another review [5]. The presence of auto-antibodies, such as RF and anti-CCP, also indicates that CHIKV may trigger autoimmunity, clinically indistinguishable from RA in non-CHIKV-infected individuals [8].

These clinical features are consistent with the known pathogenesis of post-CHIKV infection RA [14]. Viral persistence and molecular mimicry are possible mechanisms for the development of RA after CHIKV infection. Bouquillard et al. demonstrated that viral components can remain in synovial tissue long after acute infection, leading to chronic inflammation [32]. Additionally, Miner et al. [31] and Guillot et al. [30] report that CHIKV-induced arthritis shares immunological characteristics with idiopathic RA.

The efficacy of treatments for RA, such as MTX and biologic therapy in patients with CHIKV-induced arthritis has been documented in studies by Bouquillard and Combe [32] and Javelle et al. [33]. In these cohorts, patients with more severe manifestations required more aggressive treatment, such as TNF inhibitors, reflecting the chronic and refractory nature of post-CHIKV RA. These treatments significantly improved outcomes, reducing pain and disease activity. However, a recent clinical trial review did not demonstrate additional benefits of these drugs compared to anti-inflammatories or placebo [40].

Our review and meta-analysis included mostly observational studies, which may represent an important limitation because this type of study is more susceptible to selection bias and residual confounding. This may contribute to the high heterogeneity observed in the results (reduction of *I*^2^ from 96% to 76.8%), and the incidence estimate, which went from 16.2% to 12.4%. However, due to the nature of the topic investigated, there is a limited availability of randomized controlled trials that could reduce these biases. Another limitation that should be mentioned is the variation in sample sizes (between 10 and 437 patients), which may influence the precision of the pooled estimates.

Future studies should prioritize the standardization of diagnostic criteria and the long-term follow-up of specific subgroups in order to more accurately clarify the risk factors for the development of RA after CHIKV infection. Moreover, research focused on early interventions is essential to reduce the impact of this disease. Given the chronic and debilitating nature of CHIKV-associated RA, it is crucial to explore the mechanisms of autoimmunity triggered by the virus. For this, longitudinal studies with well-defined cohorts are fundamental, allowing not only for a better understanding of disease progression but also for the identification of biomarkers that facilitate early diagnosis. Finally, clinical trials are indispensable to assess the long-term efficacy and safety of biological therapies and combined treatments, offering new perspectives for therapeutic management.

## 5. Conclusions

This systematic review and meta-analysis revealed that a significant proportion of patients infected with CHIKV develop RA, with a combined estimate of 13.7% (95% CI: 6.12% to 27.87%). However, the high heterogeneity between the studies (*I*^2^ = 96%) indicates considerable variability in the findings, suggesting that factors such as differences in diagnostic criteria, study populations, and follow-up durations may have influenced the results.

These findings reinforce the need for long-term rheumatological follow-up in patients who have survived CHIKV infection, especially in endemic regions. The identification of autoimmune mechanisms associated with viral infection and the development of biomarkers for early diagnosis are important areas for future investigations.

## Figures and Tables

**Table 1:** Characteristics and outcomes of studies evaluating rheumatoid arthritis following chikungunya virus infection.

Author, Year and Country	Study Design	Nº Patients, Nº Male/FemaleAge Mean ± SD or Median (Years)	Chikungunya Diagnosis Type	Nº Rheumatoid Arthritis Diagnosed Patients	Comparators	Evaluation Measure	Description of Symptoms and Clinical Evolution	Results	Risk of Bias
Hayd et al., 2020 [20], Brazil	Cross-sectional analysis	160 patients: 40 with chronic arthritis (CHIKV), 40 without arthritis (CHIKV exposed), 40 with RA (controls), 40 healthy controls; Predominantly female; Age: 48.6 ± 11.5 (CHIKV arthritis), 43.9 ± 16.3 (CHIKV without arthritis), 51 ± 13.4 (RA Controls), 31.9 ± 11.7 (Healthy controls)	RT-qPCR confirmed CHIKV cases	4 patients	Rheumatoid arthritis controls, CHIKV exposed without arthritis, healthy controls	DAS28, EQ-5D-5L, MSQ	Initial symptoms: fever, joint pain, headache, myalgia. Chronic symptoms included persistent arthritis affecting fingers, knees, wrists, toes, and ankles, with some patients experiencing relapsing-remitting joint pain.	Over 2 years post-infection, moderate arthritis severity was reported, significantly affecting the quality of life due to pain	Moderate ^a^
Hossain et al., 2022 [22], Bangladesh	Retrospective observational cohort study	143 patients: 73 male/70 female, age: 43.3 ± 11.5	RT-PCR or IgM/IgG positive	7 patients	Patients with pre-existing arthritis (RA, SpA, OA), patients without arthritis	HAQ, RF, ACPA, X-ray sacroiliac joint, Ultrasound (musculoskeletal)	Initial symptoms included fever, polyarthralgia, symmetrical joint involvement; chronic phase symptoms included persistent mono/oligoarthritis, polyarthritis, RA, SpA.	At 1-year follow-up, 41.9% of patients had post-chikungunya chronic inflammatory rheumatism (pCHIK-CIR). Risk factors included female gender, positive IgG, and moderate to severe functional disability.	Moderate ^b^
Miner et al., 2015 [31], USA	Retrospective case series	10 patients: 3 Male/7 Female, Age range: 18–57 years	Positive for anti-CHIKV IgG (ELISA)	8 patients	none	Complete blood count, comprehensive metabolic panel, ESR, CRP, CCP, RF, ANA, CyTOF analysis of PBMCs	Acute symptoms: fever, rash, headache, symmetric polyarthritis. Chronic symptoms: persistent arthritis (hands, feet, ankles), morning stiffness, pain, joint swelling	8 of 10 patients developed RA; immune profile (T cells, NK cells) was similar to RA, highlighting diagnostic challenges.	Moderate ^c^
Tritsch et al., 2020 [21], Colombia	Cross-sectional follow-up	120 patients: 17 Male/103 Female, Age Mean: 51 ± 14 years	Clinically confirmed CHIKV, serologically confirmed (IgM and IgG)	2 patients	none	EQ-5D questionnaire, joint count, global pain score	54% of patients reported persistent joint pain 40 months post-infection; most common type of pain was relapsing-remitting; stiffness was reported by 75% of patients post-immobility.	Around 1 in 8 patients had persistent joint pain 3 years after infection. Over half of patients with joint pain experienced relapsing-remitting symptoms.	Moderate ^a^
Pollett et al., 2023 [23], USA	Retrospective cohort study	195 patients: 103 Male/92 Female; Median age: 42 years [IQR 31–54]	Diagnosed using RT-PCR or serological testing (IgM/IgG)	7 patients	Matched non-CHIKV controls (1:4 ratio) based on age, gender, beneficiary status, and healthcare encounter date	Incident rheumatic diagnoses, healthcare utilization, conditional logistic regression models	Acute symptoms included fever, polyarthralgia, backache, headache, fatigue; post-CHIKV rheumatological sequelae such as arthralgia, polyarthritis, polymyalgia rheumatica, and RA were observed.	CHIKV infection was associated with a significantly higher risk of developing rheumatological sequelae (aOR = 1.911, *p* = 0.002). 32.3% of CHIKV cases had incident rheumatic diagnoses compared to 20.0% of controls. No significant demographic, clinical, or occupational predictors were identified for rheumatic complications.	Low ^b^
Bouquillard et al., 2017 [24], France	Prospective observational cohort study	307 patients: 52 male/255 female; Mean age: 54 ± 12.6 years (24–87)	Serology (IgM and/or IgG positive)	13 patients	Patients with serological confirmation vs. clinical diagnosis; evolution of symptoms under different treatments.	HAQ score, VAS pain, synovial fluid analysis	Chronic joint pain persisted in 83.1% of patients after 32 months; synovitis observed in 64.2%, primarily affecting wrists, fingers, and ankles. Symptoms included fever, arthralgia, swollen joints, myalgia, rash, headache.	83.1% of patients reported persistent joint pain; 4% developed RA post-infection; significant functional impairment was moderate, with a mean HAQ score of 0.44 ± 0.5.	Moderate ^b^
Bouquillard and Combe, 2009 [32], France	Prospective case series	21 patients: 13 female/8 male, Mean age: 57 ± 12 years	Serology confirmed (IgM and IgG antibodies)	21 patients	none	ESR, CRP, RF, anti-CCP, radiographs, MRI	Persistent arthritis from onset of CHIKV infection; 18 with symmetric polyarthritis, 3 with oligoarthritis; high ESR, positive RF in 57.1%, anti-CCP in 28.6%, erosions in hands/feet.	Severe outcomes in most cases; radiological evidence of joint damage in 80% of patients; 6 required TNF blockers due to inadequate response to methotrexate; suggests viral role in RA initiation.	Moderate ^c^
Manimunda et al., 2010 [25], India	Prospective observational cohort study	203 patients: 96 Male/107 Female; Median age: 35 years [IQR 25–44]	Serologically confirmed (IgM antibody ELISA)	34 patients	None	ESR, X-ray, MRI, anti-CCP, RF	Acute symptoms: fever, joint pain, rash; chronic symptoms at 10 months: joint pain, swelling, fatigue; X-ray and MRI showed joint effusion, bony erosion, marrow edema, synovial thickening, tendinitis, tenosynovitis.	Chronic arthritis confirmed in 75% of patients at 10 months; 36% met ACR criteria for RA; joint erosion observed in some patients, proving that CHIK arthritis is chronic inflammatory erosive arthritis.	Low ^b^
Segura-Charry et al., 2021 [28], Colombia	Retrospective observational longitudinal study	410 patients: 47 Male/363 Female; Mean age: 57.3 ± 11.7 years	Clinical and serological confirmation (IgG)	87 patients	none	Clinical assessment by expert rheumatologists, paraclinical tests (autoantibodies, acute phase reagents), imaging studies, use of ACR/EULAR and ASAS criteria	Persistent musculoskeletal and joint symptoms for more than 3 months, with 46.3% non-inflammatory arthralgias, 21.9% post-viral polyarthritis, 20.3% de novo RA	20.3% developed RA post-CHIKV, 58.6% seropositive for RA; significant need for advanced pharmacological measures, including biological therapy in some cases.	Low ^b^
Javelle et al., 2015 [33], Reunion Island	Retrospective case series	159 patients: 40 male/119 female; Median age: 51 years (range 16–80)	Clinical and serological confirmation (RT-PCR, IgM, IgG)	40 patients	none	Time to first consultation, response to methotrexate (MTX), need for additional treatments	Persistent musculoskeletal pain, polyarthritis, spondyloarthritis; median delay of 2 years from infection to rheumatologist consultation; 66% had prolonged acute infection	MTX was effective in 75% of treated patients; hydroxychloroquine and ribavirin were ineffective; need for specific management guidelines to prevent progression to chronic disease.	Low ^c^
Amaral et al., 2018 [34], Brazil	Retrospective case series	50 patients: 4 Male/46 Female; Mean age: 61.9 ± 12.5 years	Clinical and serological confirmation (IgG serology by ELISA)	11 patients	none	VAS, Swollen and Tender Joint Count	Chronic symptoms persisted for more than 12 weeks post-infection; polyarthralgia, polyarthritis, morning stiffness, and tenosynovitis reported in many patients.	Methotrexate significantly reduced pain and disease activity; rapid reduction in VAS pain scores from baseline to 4 and 8 weeks; well-tolerated with no serious adverse events.	Moderate ^c^
Rodriguez-Morales et al., 2016 [36], Colombia	Retrospective observational cohort study	283 patients: 110 male (39%), 173 female (61%); Median age: 29 years (IQR 17–42)	Clinical confirmation and serological testing (IgM/IgG antibodies)	152 patients	None	Relative risk (RR), Kaplan-Meier survival curves, Cox regression	Persistent polyarthralgia (pCHIK-CPA) with symptoms including morning stiffness (49.5%), joint edema (40.6%), and joint redness (16.6%); higher prevalence of symptoms in patients > 40 years and females; significant persistence of symptoms up to 26 weeks.	53.7% of patients developed RA (pCHIK-CPA); more common in patients > 40 years old (69.7%) compared to ≤40 years old (47.8%); higher prevalence in females (59.5%) than males (44.5%); 19.4% of patients required ongoing medical care for persistent symptoms	Low ^b^
Ravindran and Alias, 2016 [35], India	Randomized controlled open-label clinical trial	72 patients: 37 in combination therapy, 35 in monotherapy; Combination group: 4 male, 33 female; Monotherapy group: 5 male, 30 female; Age Mean ± SD: Combination: 54.05 ± 6.67, Monotherapy: 56.6 ± 7.64	Serologically confirmed (virus-specific IgM antibodies)	12 patients	Combination therapy (MTX, SSZ, and HCQ) vs. HCQ monotherapy	DAS28, HAQ, VAS	Persistent arthritis for >1 year post-CHIKV infection; symptoms included swollen joints, tenderness, and high disease activity. Both groups were previously on HCQ with active disease; assessed for response to combination therapy versus monotherapy.	Combination therapy significantly improved DAS28, HAQ, and VAS compared to monotherapy; 85% achieved EULAR good response in combination group vs. 14% in monotherapy group; combination therapy more effective in achieving low disease activity and reducing pain.	Some concerns ^d^
Paul et al., 2011 [26], India	Prospective observational cohort study	150 patients: 40 male/110 female; Age range: 20–60 years	Serological confirmation (IgM capture ELISA)	5 patients	None	Symptom duration, response to antimalarials and steroids	100% of patients had arthralgia, 78% had arthritis, 22% had persistent symptoms beyond one year; common symptoms included fever, rash, and morning stiffness; antimalarials and short courses of steroids were partially effective in some cases.	22% developed chronic symptoms; 5% of patients developed RA after CHIKV infection; RA cases had anti-CCP positivity; synovial biopsies showed chronic inflammation.	Low ^b^
Mathew et al., 2011 [27], India	Retrospective observational cohort study	437 patients: 124 male/313 female; Mean age: 48.37 ± 13.62 years	Clinical confirmation and serology (IgM antibodies)	6 patients	None	Pain site assessment, ACR criteria for rheumatic diseases, lab tests (ESR, CRP, RF, anti-CCP), musculoskeletal ultrasound	Common pain sites: knee (83.3%), ankle (63.2%), lower back (49.4%); 57% had post-viral polyarthralgia, 22% had post-viral polyarthritis.	8.3% incidence of RMSK pain in naïve group; higher prevalence of chronic rheumatic disorders post-CHIKV, including RA and seronegative spondyloarthritis.	Low ^b^
Guillot et al., 2020 [30], Reunion Island	Retrospective observational cohort study	159 patients: Gender distribution not fully specified; Age data not provided	Clinical confirmation and serological testing (IgM/IgG antibodies)	40 patients	none	Clinical examination, autoantibodies (ACPA, RF, ANA), radiographic evaluations, DAS28 score, BASDAI score	Inflammatory joint symptoms persisted after 13 years; higher prevalence of positive autoantibodies (antinuclear or ACPA) without seroconversion, and radiographic joint damage in a subgroup	23.3% of patients initially diagnosed with CHIK-related inflammatory joint symptoms continued to have symptoms after 13 years; long-term IgM positivity and radiographic damage were more frequent in the persistence group.	Low ^b^

ANA: antinuclear antibody; BASDAI: bath ankylosing spondylitis disease activity index; CCP: cyclic citrullinated peptide; CHIKV: chikungunya virus; CRP: C-reactive protein; CyTOF: cytometry by time-of-flight; DAS28: Disease Activity Score in 28 joints; EQ-5D-5L: The EuroQol 5-Dimension 5-level; ESR: erythrocyte sedimentation rate; HAQ: Health Assessment Questionnaire; MRI: magnetic resonance imaging; MSQ: musculoskeletal stiffness questionnaire; OA: osteoarthritis; PBMCs: peripheral blood mononuclear cells; RA: rheumatoid arthritis; RF: rheumatoid factor; RMSK: rheumatic-musculoskeletal; RT-qPCR: reverse transcription-quantitative (polymerase chain reaction); SpA: spondyloarthritis; TNF: tumor necrosis factor; VAS: visual analogue scale. ^a^: Based on The Joanna Briggs Institute Critical Appraisal tool for cross-sectional analysis studies. ^b^: Based on The Joanna Briggs Institute Critical Appraisal tool for cohort studies. ^c^: Based on the Newcastle–Ottawa Scale (NOS) tool for case series. ^d^: Based on RoB 2.0 tool for randomized trials.

## Data Availability

The original contributions presented in this study are included in the article and Supplementary Materials. Further inquiries can be directed to the corresponding author.

## Supplementary Materials

The following supporting information can be downloaded at: https://www.mdpi.com/article/10.3390/tropicalmed10020054/s1, Table S1: Details of risk of bias assessments based on the Newcastle–Ottawa Scale (NOS) for cohort studies; Table S2: Details of risk of bias assessments based on The Joanna Briggs Institute Critical Appraisal tool for cross-sectional analysis studies; Table S3: Details of risk of bias assessments based on The Joanna Briggs Institute Critical Appraisal tool for cohort studies; Table S4: Details of risk of bias assessments based on The Joanna Briggs Institute Critical Appraisal tool for case series; Table S5: Details of risk of bias assessments based on RoB 2.0 tool for randomized trials.
