# Supplementary material for: Chikungunya Fever and Rheumatoid Arthritis: A Systematic Review and Meta-Analysis
Source: Trop Med Infect Dis. 2025 Feb 12;10(2):54. doi: 10.3390/tropicalmed10020054 (PMC11861052; doi:10.3390/tropicalmed10020054)
Supplement: Supplementary file 1 [file tropicalmed-10-00054-s001.zip › tropicalmed-3453195-supplementary.pdf]

Supplementary Table 1. Details of risk of bias assessments based on the Newcastle-Ottawa Scale (NOS) for cohort studies.

Pollett et al., 2023, USA		
Category	**Criteria**	
Selection		
1) Representativeness of the exposed cohort	a) Truly representative of the average _______________ in the community	★
	b) Somewhat representative of the average ______________ in the community	
	c) Selected group of users, e.g., nurses, volunteers	
	d) No description of the derivation of the cohort	
2) Selection of the non-exposed cohort	a) Drawn from the same community as the exposed cohort	★
	b) Drawn from a different source	
	c) No description of the derivation of the non-exposed cohort	
3) Ascertainment of exposure	a) Secure record (e.g., surgical records)	★
	b) Structured interview	
	c) Written self-report	
	d) No description	
4) Demonstration that outcome of interest was not present at start of study	a) Yes	★
	b) No	
Comparability		
1) Comparability of cohorts on the basis of the design or analysis	a) Study controls for **age, sex, beneficiary status, and pre-existing rheumatic diseases**	★★
	b) Study controls for any additional factor (this criterion could be modified to indicate specific control for a second important factor)	
Outcome		
1) Assessment of outcome	a) Independent blind assessment	
	b) Record linkage	★
	c) Self-report	
	d) No description	
2) Was follow-up long enough for outcomes to occur	a) Yes (select an adequate follow-up period for outcome of interest)	★
	b) No	
3) Adequacy of follow-up of cohorts	a) Complete follow-up - all subjects accounted for	
	b) Subjects lost to follow-up unlikely to introduce bias - small number lost - > ____ % (select an adequate %) follow-up, or description provided of those lost	
	c) Follow-up rate < ____% (select an adequate %) and no description of those lost	
	d) No statement	★

Supplementary Table 2. Details of risk of bias assessments based on The Joanna Briggs Institute Critical Appraisal tool for cross-sectional analysis studies.

Study	*Hayd et al. (2020, Brazil)*	*Tritsch et al. (2020, Colombia)*
1. Were the criteria for inclusion in the sample clearly defined?	Yes	Yes
2. Were the study subjects and the setting described in detail?	Yes	Yes
3. Was the exposure measured in a valid and reliable way?	Yes	Yes
4. Were objective, standard criteria used for measurement of the condition?	Yes	Yes
5. Were confounding factors identified?	Yes	Yes
6. Were strategies to deal with confounding factors stated?	Unclear	No
7. Were the outcomes measured in a valid and reliable way?	Yes	Yes
8. Was appropriate statistical analysis used?	Yes	Yes

Supplementary Table 3. Details of risk of bias assessments based on The Joanna Briggs Institute Critical Appraisal tool for cohort studies.

Study	*Q1*	*Q2*	*Q3*	*Q4*	*Q5*	*Q6*	*Q7*	*Q8*	*Q9*	*Q10*	*Q11*
Hossain et al. (2022)	NA	NA	Yes	Yes	Yes	Yes	Yes	Yes	Unclear	Unclear	Yes
Bouquillard et al. (2017)	NA	NA	Yes	Yes	No	Yes	Yes	Yes	Unclear	No	Yes
Manimunda et al. (2010)	NA	NA	Yes	Yes	Yes	Yes	Yes	Yes	Yes	Yes	Yes
Segura-Charry et al. (2021)	NA	NA	Yes	Yes	Yes	Yes	Yes	Yes	Yes	Yes	Yes
Rodriguez-Morales et al. (2016)	NA	NA	Yes	Yes	Yes	Yes	Yes	Yes	Yes	Yes	Yes
Paul et al. (2011)	NA	NA	Yes	Yes	Yes	Yes	Yes	Yes	Yes	Yes	Yes
Mathew et al. (2011)	NA	NA	Yes	Yes	Yes	Yes	Yes	Yes	Yes	Yes	Yes
Guillot et al. (2020)	NA	NA	Yes	Yes	Yes	Yes	Yes	Yes	Yes	Yes	Yes

NA: not applicable

Q1. Were the two groups similar and recruited from the same population?

Q2. Were the exposures measured similarly to assign people to both exposed and unexposed groups?

Q3. Was the exposure measured in a valid and reliable way?

Q4. Were confounding factors identified?

Q5. Were strategies to deal with confounding factors stated?

Q6. Were the groups/participants free of the outcome at the start of the study (or at the moment of exposure)?

Q7. Were the outcomes measured in a valid and reliable way?

Q8. Was the follow-up time reported and sufficient to be long enough for outcomes to occur?

Q9. Was follow-up complete and if not, were the reasons for loss to follow-up described and explored?

Q10. Were strategies to address incomplete follow-up utilized?

Q11. Was appropriate statistical analysis used?

Supplementary Table 4. Details of risk of bias assessments based on The Joanna Briggs Institute Critical Appraisal tool for case series.

Study	*Q1*	*Q2*	*Q3*	*Q4*	*Q5*	*Q6*	*Q7*	*Q8*	*Q9*	*Q10*
Miner et al. (2015)	Yes	Yes	Yes	Unclear	Unclear	Yes	Yes	Yes	Yes	Yes
Bouquillard and Combe (2009)	Yes	Yes	Yes	No	Yes	Yes	Yes	Yes	Yes	Yes
Javelle et al. (2015)	Yes	Yes	Yes	Yes	Yes	Yes	Yes	Yes	Yes	Yes
Amaral et al. (2018)	Yes	Yes	Yes	No	Yes	Yes	Yes	Yes	Yes	Yes

Q1: Were there clear criteria for inclusion in the case series?

Q2: Was the condition measured in a standard, reliable way for all participants included in the case series?

Q3: Were valid methods used for identification of the condition for all participants included in the case series?

Q4: Did the case series have consecutive inclusion of participants?

Q5: Did the case series have complete inclusion of participants?

Q6: Was there clear reporting of the demographics of the participants in the study?

Q7: Was there clear reporting of clinical information of the participants?

Q8: Were the outcomes or follow up results of cases clearly reported?

Q9: Was there clear reporting of the presenting site(s)/clinic(s) demographic information?

Q10: Was statistical analysis appropriate?

Supplementary Table 5. Details of risk of bias assessments based on RoB 2.0 tool for randomized trials.

Ravindran and Alias (2016).

Bias Domain	*Signaling Questions*	*Response Options*	*Risk-of-Bias Judgment*	*Comments*
Domain 1: Bias arising from the randomization process	1.1 Was the allocation sequence random?<br>1.2 Was the allocation sequence concealed?<br>1.3 Did baseline differences suggest a problem?	Yes<br>Probably yes<br>No	Low risk	Randomization was done using a computer-generated sequence; allocation was concealed using sealed envelopes; baseline characteristics were well balanced.
Domain 2: Bias due to deviations from intended interventions	2.1 Were participants aware?<br>2.2 Were carers aware?<br>2.3 Were there deviations?<br>2.4 Were deviations likely to affect the outcome?<br>2.5 Were deviations balanced?<br>2.6 Was analysis appropriate?<br>2.7 Was there potential impact from analysis failure?	Yes<br>Yes<br>No<br>Not applicable<br>Not applicable<br>Yes<br>Not applicable	Low risk	The study was open-label; no deviations due to trial context; appropriate intention-to-treat analysis was conducted. Questions 2.4 and 2.5 are not applicable because no deviations were identified. Question 2.7 is not applicable as the analysis was appropriate.
Domain 3: Bias due to missing outcome data	3.1 Were outcome data available for most participants?<br>3.2 Was there evidence result not biased by missing data?<br>3.3 Could	Yes<br>Not applicable<br>Not applicable<br>Not applicable	Low risk	Outcome data were available for nearly all participants, hence questions 3.2, 3.3, and 3.4 are not applicable.

	missingness depend on its true value?<br>3.4 Likelihood missingness depended on its true value?			
Domain 4: Bias in measurement of the outcome	4.1 Was the method of measuring inappropriate?<br>4.2 Could measurement differ?<br>4.3 Were assessors aware?<br>4.4 Could assessment be influenced?<br>4.5 Is it likely that assessment was influenced?	No<br>No<br>Yes<br>Probably yes<br>Yes	Some concerns	Open-label design could have influenced subjective outcomes due to knowledge of intervention received.
Domain 5: Bias in selection of the reported result	5.1 Was the analysis pre-specified?<br>5.2 Was result selected based on multiple measurements?<br>5.3 Was result selected based on multiple analyses?	Yes<br>No<br>No	Low risk	The analysis was pre-specified and adhered to the plan.

Overall risk-of-bias judgment: Some concerns due to potential bias in the measurement of the outcome, particularly because of the open-label design, where knowledge of the intervention received could have influenced the outcome assessment.
